# Rethinking the Process of Animal Mummification in Ancient Egypt: Molecular Characterization of Embalming Material and the Use of Brassicaceae Seed Oil in the Mummification of Gazelle Mummies from Kom Mereh, Egypt

**DOI:** 10.3390/molecules27051532

**Published:** 2022-02-24

**Authors:** Milan Marković, Elodie Mezzatesta, Stéphanie Porcier, Cathy Vieillescazes, Carole Mathe

**Affiliations:** 1 IMBE UMR 7263, IRD237, Avignon University/CNRS/IRD/Aix-Marseille University, Restoration Engineering of Natural and Cultural Heritage, Faculty of Sciences, Campus Jean-Henri Fabre, 301 rue Baruch de Spinoza BP 21239, CEDEX 9, 84916 Avignon, France; elodie.mezzatesta@univ-avignon.fr (E.M.); cathy.vieillescazes@univ-avignon.fr (C.V.); 2 ASM, Archéologie des Sociétés Méditerranéennes, UMR5140, Centre National de la Recherche Scientifique, Universités Paul Valéry, MCC (Ministère de la Culture et de la Communication), INRAP (Institut National de Recherches en Archéologie Préventive), CEDEX 5, F-34090 Montpellier, France; stephanie.porcier@gmail.com

**Keywords:** gazelle mummies, mummification balms, Brassicaceae seed oil, Roman period Egypt, infrared spectroscopy, gas chromatography, mass spectrometry

## Abstract

The study of animal mummification in ancient Egypt has recently received increasing attention from a number of modern scholars given the fact that this part of ancient Egyptian funerary and religious history is a practice yet to be fully understood. In this study, nine samples of embalming matter were extracted from six gazelle mummies from the archaeological site of Kom Mereh (modern village of Komir), dated to the Roman period of dominance in ancient Egypt. All samples were analyzed for the presence of inorganic and organic matter applying a multi-analytical approach based on Fourier transform infrared spectroscopy (FT-IR) and gas chromatography–mass spectrometry (GC-MS). Furthermore, in order to identify more specific compounds such as bitumen and beeswax in studied balms, each sample was subjected to a solid phase extraction (SPE) and saponification separation process, respectively. The results of this study revealed that the majority of the analyzed embalming substances sampled from six gazelle mummies from Kom Mereh were complex mixtures of plant oils, animal fats, conifer resin, and beeswax. In this regard, this study was able to report a practice until now unmentioned in the scientific literature, namely, the use of cruciferous oil, derived from seeds of Brassicaceae plants, in animal mummification.

## 1. Introduction

In the context of studying the cultural, economic, political, and religious past of ancient Egypt, the use of a cruciferous oil derived from the seeds of specific Brassicaceae species (such as radish and/or rapeseed), is mentioned in written historical sources, mostly in the works of the Roman historian Pliny (*Naturalis Historia*, books XV:7 and XIX:26) during the 1st century AD, and in several 5th century AD papyri documents (P.Mich XI: 613). In these historical records, different classical authors refer to the use of radish oil exclusively, either as a fuel for oil lamps or as a staple food, and according to these texts, it was one of the most common oil crop commodities in Roman Egypt, due to its preferable properties such as the quantity of produced product compared to sesame, castor and other oils, its nutritional value, and most importantly, lower taxes [1]. Furthermore, several archaeometric studies in the past 15 years confirmed the writings of the above-mentioned historical records. Specifically, in the works of M. Colombini [2,3] and M.S. Copley [4], it was reported that in analyzed ceramic lamps from the sites of Antinoe (5th to 8th century AD) and Qasr Ibrim (6th century AD) in Roman Egypt, the detection of radish oil as an illuminant was demonstrated through the presence of diagnostic and characteristic molecular biomarkers. Moreover, results published in the paper by Kerlijne Romanus [5] also indicated that organic residues preserved in eight lamp shells from Coptic site Bawit (8th to 10th century AD) were from seed oils derived from Brassicaceae plants (authors specifically indicate the presence of *Raphanus sativus* (radish)) and, compared to previous cases, were used as an illuminant. To summarize, up to this date in classical literary sources and contemporary scientific studies, the only data regarding the use of Brassicaceae seed oil in the context of ancient Egypt are related to its use as an illuminative agent or as a dietary supplement.

Hence, for the first time in a narrow scientific community engaged in the study of animal mummification, the authors of this paper argue that cruciferous oil obtained from the seeds of Brassicaceae plants could also be used as an embalming agent in the context of mummification practices in Roman Egypt. If we take into account the fact that, indeed, there is no mention of oils obtained from the seeds of Brassicaceae plants in the context of animal mummification in ancient Egypt, but rather as a means of illumination, the importance of the present study is twofold: (i) providing valuable insights about the possibilities of molecular analyses in identifying complex molecular compositions of new and unknown ingredients, and (ii) contributing new datasets comparable to the already existing, but narrow, corpus of knowledge concerning organic substances used for animal mummification [6,7,8,9].

To this end, of particular interest for this study were mummified remains of six votive gazelle mummies as part of an Egyptology collection from the Confluence Museum in Lyon (France), which had been collected in the early 20th century by Dr. Louis Lortet (rector of the Natural History Museum of Lyon at that time) and French naturalist Claude Gaillard [10]. Furthermore, these animal mummies were dated to Roman Egypt, and they were discovered during one of the excavation campaigns carried out in 1882 near the village of Kom Mereh/Komir [11]. Collected archaeological data suggested that these mummified remains of gazelles were associated with the local aspect of the goddess Anukis-Nephthys, which is in accordance with historical records that connected this deity to the city of Komir as early as the New Kingdom and up to Roman times [12,13].

In this paper, spectroscopic and chromatographic analytical techniques were employed to investigate the molecular composition of complex mixtures, such as embalming substances. In this way, Fourier transform infrared spectroscopy (FT-IR) and gas chromatography coupled with mass spectrometry (GC-MS) were used as complementary techniques. This research work aimed to identify the ingredients in nine sample extracts used for mummification that were recovered from six gazelle mummies, at the same time inquiring about the “recipe/recipes” that were applied in the preparation of the final product and in what (possible?) way the ingredients could have been admixed. Therefore, in this way, the obtained information allowed us to place gazelles from Kom Mereh in a narrower scientific context of animal mummification in Roman Egypt, as a result of comparative analysis of data obtained from several published works on other animal mummies from this time period.

## 2. Results and Discussion

The analyzed samples were in most cases organic in nature, with inorganic traces in addition to organic residues detected in only a few gazelle mummies. Based on obtained data, together with an interpretation of diagnostic infrared bands from FT-IR and molecular biomarker components recovered from GC-MS, it was safe to presume four main groups of families were used as ingredients to produce balms that were later applied in animal mummification: plant oil, animal fat, conifer resin, and beeswax. More detailed results in terms of identified functional groups and bond assignments can be seen in Table 1, where FT-IR data are provided, while in Table 2, Table 3 and Table 4, the results of various GC-MS data treatments are given.

### 2.1. FT-IR

Results showed that IR spectra were consistent within an individual mummy regardless of the place where the sample was taken; in other words, data did not show any significant or major deviations in identified components, although absorption intensity might vary, which was expected. The only exception to this claim was the case of gazelle mummy 90002284, where FT-IR data showed minor differences between sample extracted from the interior part of the chest area (a) and textile recovered from the head of the mummy (b) bundle (Table 1). The infrared spectra of all gazelle mummies analyzed in this paper showed bands characteristic of organic matter present in the studied balm, with the addition of samples 90002282 and 90002283 displaying bands diagnostic for inorganic matter as well (Table 1). Regarding the inorganic content diagnosed in samples 90002282 and 90002283, strong bands at ~2519 cm^−1^ (stretching of O-H bond of HCO_3_ in bicarbonates), 1427 cm^−1^ (stretching of O-H bond in carbonates), and 874 cm^−1^ and 712 cm^−1^ (out-of-plane and in-plane bending of C-O bonds in carbonates) unambiguously indicated the presence of carbonates in the studied specimens [14,15,16,17]. Unequivocally, this result served as a direct indicator of the use of either natron (sodium carbonate) or lime (calcium carbonate) during the mummification process of these two gazelle mummies from Kom Mereh. However, considering that these two animal mummies originated from the Roman era of dominance in ancient Egypt, it can be argued that sodium carbonate was used, as it is one of the main components of natron salt, considering that from the New Kingdom period onward, natron was predominantly used as proven by many studies, either archaeometric or from experimental archaeology [18]. The use of natron (or lime) in the process of mummification in ancient Egypt was practiced not only because of its dehydrating abilities but also because of its natural antibacterial and deodorizing properties [18,19]. In both samples, a strong band emitting at ~1318 cm^−1^ suggested the presence of calcium oxalates, which are common components of degraded organic materials [7]. This particular band was also present in all six gazelle mummies. However, precise determination of the botanical origin was challenging due to their quite ubiquitous nature. Calcium oxalates can be found in all major groups of photosynthetic organisms, including algae, lower vascular plants, gymnosperms, and angiosperms, and they are particularly abundant in these last two groups, although not all plants produce them. In addition to this, calcium oxalates can be induced by the metabolic reaction of microorganisms that could promote the occurrence of oxalates from the degraded organic matter they infiltrated. To summarize, calcium oxalates cannot be used as a biomarker for any specific organic material due to their ubiquitous nature [20,21,22,23].

**Figure 1:**
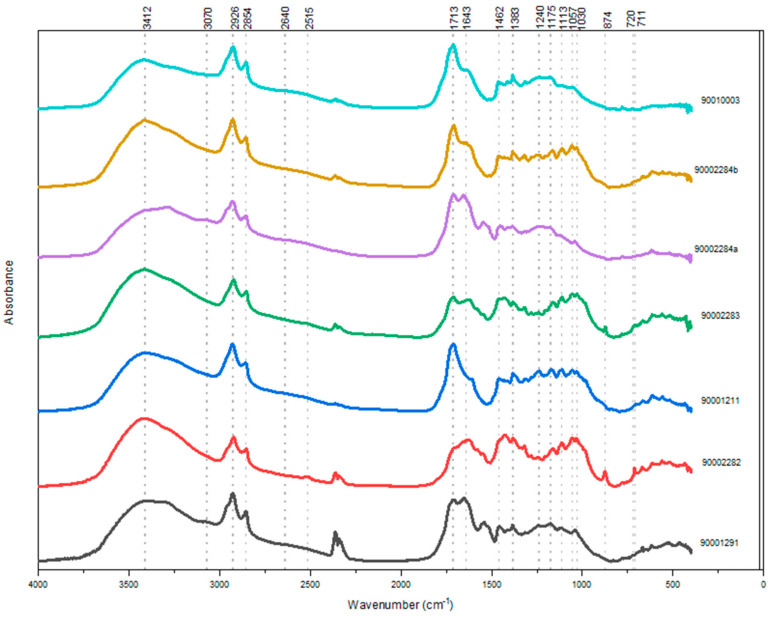
Infrared spectra of analyzed balms sampled from six gazelle mummies from Kom Mereh.

As mentioned before in this paper, all analyzed samples indicated a strong presence of organic matter (Figure 1) through diagnostic and specific infrared bands characteristic for hydroxyl (~3400 cm^−1^), methyl/methylene (~2960–2850 cm^−1^), and carbonyl (~1750–1650 cm^−1^) functional groups [2,6,7,14,24]. In this regard, further assessment of the obtained results indicated that infrared spectra of all samples demonstrated a standard transmittance profile specific for aged diterpenoid resins extracted from the Pinaceae family [6,24,25]. With this in mind, the most prominent diagnostic infrared bands of this type of natural (aged) substance, and also present in all analyzed samples, were observed at 2964–2855 cm^−1^ (the result of symmetric and asymmetric stretching of CH_3_ and CH_2_ functional groups from hydrocarbon skeletons present in tricyclic diterpenic structures) along with methyl and methylene group bending vibrations at 1384–1383 cm^−1^ and 1465–1454 cm^−1^, and 1715–1705 cm^−1^ (carbonyl stretching in ester and/or carboxyl functional groups) [7,14,24,26,27,28,29,30]. Indeed, the presence of the carbonyl band would rather indicate the presence of triterpenoids [7,27], but such a scenario is very difficult to confirm, especially, in the case of archaeological artifacts, notably if it is a mixture of substances such as balms used in animal mummification where one must anticipate wavelength shift due to several internal/external factors [6,15,27]. On the other hand, in samples 90001291, 90002284, and 90010003, the bands around 3200 and 2600 cm^−1^ corresponded to O-H stretching, overtones, and combined bands that, according to reference spectra in published literature, could indicate the presence of diterpenoid resins extracted from coniferous trees such as fir and pine [2,24,28]. Furthermore, in samples 90001291 and 90002284 we could observe a weak band around 3070 cm^−1^ that corresponded to stretching vibration of C=C double bonds characteristic for diterpenoids with (possible) pimarane skeletons [24,28,29]. In addition to these data, in all samples, absorption bands centered around ~1243 cm^−1^ and ~1175 cm^−1^ were bending signals from COOH groups present in excessively oxidized chemical structures that can be found in aged and thermally degraded conifer resins used by ancient Egyptians [6,24,25]. Indeed, natural resins, such as those obtained from conifers, could undergo oxidation, degradation, and alteration processes naturally over time (aging) or could be a result of anthropogenic degradation, meaning that the resin was subjected to an intense heating process in order to achieve a certain property of the material. Considering these identified diagnostic frequencies present in the studied samples, it is safe to acknowledge the presence of a mixture of oxidized and dehydrogenated molecules that are normally found in pine resins.

Taking the obtained FT-IR results into account, we can consider the possibility of using coniferous resins from the Pinaceae family of trees as one of the ingredients in the embalming recipe used for the mummification of these gazelle mummies. Regarding the remaining identified substances in the analyzed samples, absorption bands around 1609–1637 cm^−1^ corresponded to stretching vibration of double C=C bonds that could be due to the presence of polysaccharides. Precise determination will be a priority for future research in terms of defining whether this band could be a result of the presence of sugars in plant gums in the original embalming recipe or signals originated from the linen textiles in which the embalmed animals were wrapped [31]. Additionally, fatty matter in studied balms in the form of plant oils and/or animal fats in samples 90001291, 90002282, 90001211, 90002283, 90002284a/b, and 90010003 were observed through absorption bands reflecting the presence of rocking C-H bands at ~720 cm^−1^ and various stretching C-O bands from ~1175–1161 cm^−1^. These diagnostic bands could indicate the presence of long linear molecular chains of fatty acids present in plant oils and animal fats. What is very important to point out at this moment is the fact that based on the presented results obtained by FT-IR analysis, it is possible to assume the presence of beeswax in the analyzed samples according to diagnostic bands of beeswax known from the literature (two strong and sharp bands at 2920 and 2850 cm^−1^, C=O band around ~1720 cm^−1^ reflecting ester function, rocking C-H band at 720 cm^−1^). However, bearing in mind that all of these bands could also be from other organic substances present in the analyzed samples (especially from mixtures such as ancient Egyptian embalming substances), such as plant oils and/resins, this hypothesis will be either confirmed or rejected by GC-MS results.

### 2.2. GC-MS

**Figure 2:**
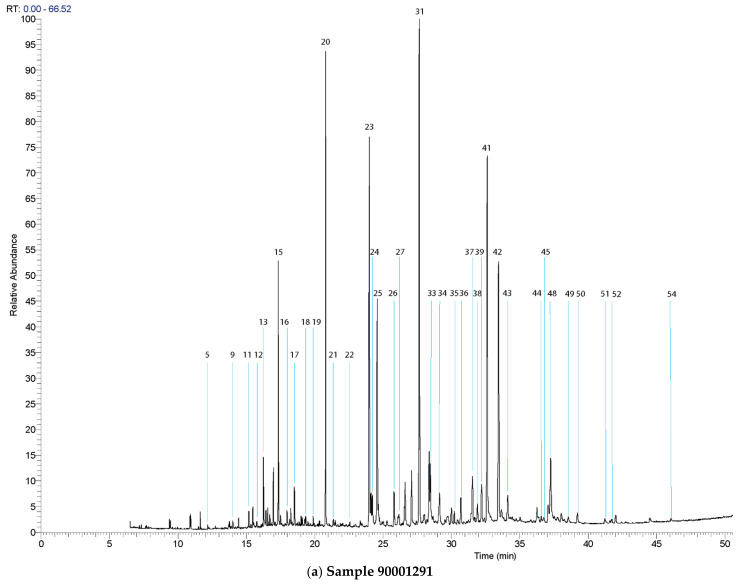

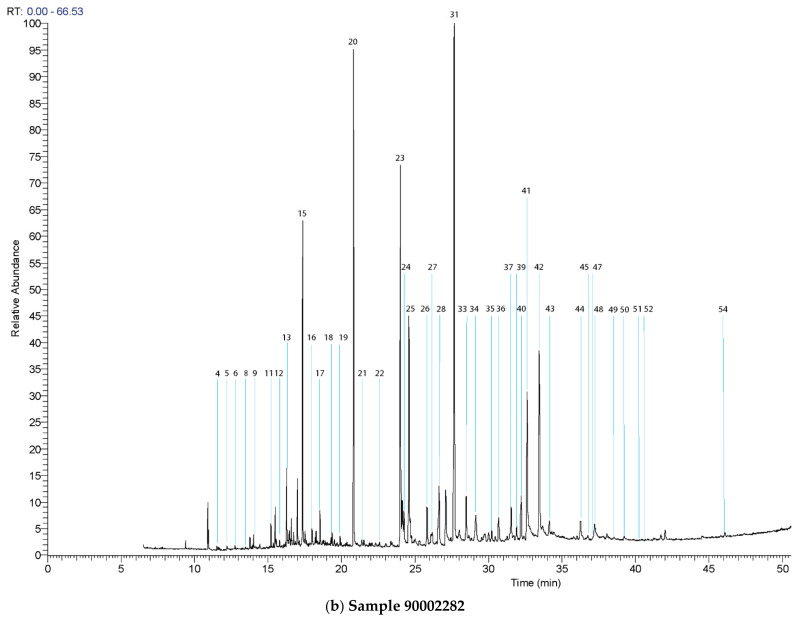
Partial total ion current chromatograms of samples 90001291 (**a**) and 90002282 (**b**). All of the compounds were identified as their TMS derivatives.

As far as further research is concerned, the analyzed data provided the necessary input essential for observing the chromatographic patterns, which in several cases, regardless of the place from where the sample was extracted, proved almost identical (within individual animals), although the relative abundance varied, albeit insignificantly. Bearing in mind that these animals lived in the same chronological period (Roman times) and originated from the same archaeological site (Kom Mereh in Upper Egypt), it is safe to assume that they were mummified in the same manner with highly similar “recipes”. Figure 2 represents the obtained chromatograms for samples 90001291 and 90002282, respectively, while in Table 2, Table 3 and Table 4, the results of the GC-MS analysis are summarized in the form of a list of identified compounds in all samples that were the focus of this paper. In addition to the aforementioned data, GC-MS results unambiguously showed that the primary commodities used for mummification, present in analyzed balms, originated from a highly complex mixture of plant oils, animal fats, plant resins, and, in certain cases, beeswax.

#### 2.2.1. Presence of Plant Oils and Animal Fats

Preliminary results based on the interpretation of chromatograms of nine samples extracted from six gazelle mummies revealed several different types of characteristic compounds that unequivocally indicated the presence of both plant oil and animal fat in the original embalming material, thus confirming the fact that these commodities were essential parts during the mummification process of these creatures. In this regard, in chromatograms of samples 90001211, 90002283 and 90010003, the presence of odd carbon-numbered, straight-chain compounds, specifically, C_15:0_ (pentadecanoic acid, M°^+^ 314 and diagnostic fragment ions of *m*/*z* 75, 117, 299) and C_17:0_ (heptadecanoic acid, M°^+^ 342 and diagnostic fragment ions of *m*/*z* 75, 117, 327), served as good indicators of the presence of ruminant animal fat, thus implying it was one of the ingredients used for embalming purposes (Table 2). As already thoroughly explained in previous research works by Eerkens [32] and Markovic et al. [6], by calculating the ratio of the sum of the peak areas of corresponding odd and even ((C_15:0_ + C_17:0_)/(C_12:0_ + C_14:0_ + C_16:0_ + C_18:0_)) chain fatty acids, it is possible to argue whether the analyzed samples could contain traces of lipids from animal fats when the result is higher than 0.04 (Table 3) [9,32,33,34,35]. Even though this result was quite compelling, we should be aware of the fact that the odd carbon long-chain compounds observed in the corresponding GC-MS chromatograms also could be due to microbial contamination. Most probably, future research works with the introduction of gas chromatography-combustion-isotope ratio mass spectrometry (GC-C-IRMS) will clarify this situation with the addition of a potential determination of which animal the given fat was made for the purpose of mummification. This topic has been widely discussed on numerous occasions by several different authors who have studied ancient Egyptian mummification, although with the bodies of deceased people [9,33,34,35,36,37]. On the other hand, in chromatograms of all nine analyzed samples extracted from six gazelle mummies, the fatty-acid distribution profile indicated the presence of plant oil (higher value of C_16:0_ compared to C_18:0_, presence of unsaturated fatty acids, range of dicarboxylic acids, etc.). However, when we consider that many of the plant oils mentioned in ancient literature that were used in ancient Egyptian mummification practices have almost identical composition, followed by the fact that in the majority of cases they were part of complex and aged/degraded mixtures with resulting molecular patterns, in the end represent an aggravating circumstance when it comes to the precise determination of the exact botanical source of the original material. Nevertheless, in rare and special cases, it is possible to argue about the exact origin of the plant from which the oil was produced for mummification purposes, yet only when the characteristic biomarkers have been preserved. A good example of this would be the presence of high amounts of 12-hydroxy-9-*cis*-octadecenoic acid, which serves as a diagnostic compound for the presence of castor oil obtained from the seeds of Ricinus communis L. (Euphorbiaceae), as ricinoleic acid is not found in other natural lipids [3,6,9,14,15,31,38].

**Table 2:** List of compounds identified by GC-MS analysis of embalming substances from gazelle mummies (D: dicarboxylic acids; S: Saturated fatty acids; U: Unsaturated fatty acids; R: Resinic acids; DH: Dihydroxycarboxylic acids; St: Sterols).

No	Class	Identified Compounds	Samples					
			90001291	90002282	90001211 *	90002283	90002284 *	90010003
1	S	Caproic acid (C6:0)	-	-	**✓** (b)	-	**✓**	**✓**
2	S	Enanthic acid (C7:0)	-	-	**✓**	**✓**	**✓**	**✓**
3	S	Caprylic acid (C8:0)	-	-	**✓**	**✓**	**✓**	**✓**
4	D	Succinic acid	-	**✓**	**✓**	**✓**	**✓**	**✓**
5	S	Pelargonic acid (C9:0)	**✓**	**✓**	**✓**	**✓**	**✓**	**✓**
6	D	Glutaric acid	-	**✓**	**✓**	**✓**	**✓** (b)	**✓**
7	U	Caproleic acid (C10:1)	-	-	-	-	-	-
8	S	Capric acid (C10:0)	-	**✓**	**✓**	**✓**	**✓**	**✓**
9	D	Adipic acid	**✓**	**✓**	**✓**	**✓**	**✓**	**✓**
10	S	Undecylic acid (C11:0)	-	-	**✓** (a)	**✓**	**✓**	**✓**
11	D	Pimelic acid	**✓**	**✓**	**✓**	**✓**	**✓**	**✓**
12	S	Lauric acid (C12:0)	**✓**	**✓**	**✓**	**✓**	**✓**	**✓**
13	D	Suberic acid	**✓**	**✓**	**✓**	**✓**	**✓**	**✓**
14	S	Tridecylic acid (C13:0)	-	-	-	**✓**	**✓** (b)	**✓**
15	D	Azelaic acid	**✓**	**✓**	**✓**	**✓**	**✓**	**✓**
16	S	Myristic acid (C14:0)	**✓**	**✓**	**✓**	**✓**	**✓**	**✓**
17	D	Sebacic acid	**✓**	**✓**	**✓**	**✓**	**✓**	**✓**
18	S	Pentadecanoic acid (C15:0)	**✓**	**✓**	**✓**	**✓**	**✓**	**✓**
19	D	1,11-Undecanedioic acid	**✓**	**✓**	**✓**	**✓**	**-**	**-**
20	S	Palmitic acid (C16:0)	**✓**	**✓**	**✓**	**✓**	**✓**	**✓**
21	D	Dodecanedioic acid	**✓**	**✓**	**✓**	**✓**	**-**	**-**
22	S	Margaric acid (C17:0)	**✓**	**✓**	**✓**	**✓**	**✓**	**✓**
23	U	Oleic acid (C18:1)	**✓**	**✓**	**✓**	**✓**	**✓**	**✓**
24	R	Retene	**✓**	**✓**	**✓**	**✓**	**✓**	**✓**
25	S	Stearic acid (C18:0)	**✓**	**✓**	**✓**	**✓**	**✓**	**✓**
26	R	Pimaric acid	**✓**	**✓**	**✓** (a)	-	**✓**	**✓**
27	R	Sandaracopimaric acid	**✓**	**✓**	**✓** (a)	-	**✓**	**✓**
28	R	Isopimaric acid	**✓**	**✓**	**✓** (a)	-	**✓**	**✓**
29	R	Methyl dehydroabietate	**✓**	**✓**	**✓** (a)	-	-	-
30	R	Palustric acid	-	-	-	-	-	**✓**
31	R	Dehydroabietic acid	**✓**	**✓**	**✓**	**✓**	**✓**	**✓**
32	R	Abietic acid	-	-	-	-	-	**✓**
33	U	Gondoic acid (C20:1)	**✓**	**✓**	**✓**	**✓**	**✓**	**-**
34	S	Arachidic acid (C20:0)	**✓**	**✓**	**✓**	**✓**	**✓**	**✓**
35	R	3-hydroxy-dehydroabietic acid	**✓**	**✓**	**✓**	**✓**	**✓**	**✓**
36	R	7-hydroxy-dehydroabietic acid	**✓**	**✓**	**✓**	**✓**	**✓**	**✓**
37	DH	9,10-dihydroxyoctadecanoic acid	**✓**	**✓**	**✓** (b)	**✓**	**✓**	**✓**
38	R	15-Hydroxy-dehydroabietic acid	**✓**	**✓**	-	-	-	-
39	DH	9,10-dihydroxyoctadecanoic acid	**✓**	**✓**	**✓** (b)	**✓**	**✓**	**✓**
40	R	7-oxo-dehydroabietic methyl ester	**✓**	**✓**	**✓** (a)	**✓**	**✓**	**✓**
41	R	7-oxo-dehydroabietic acid	**✓**	**✓**	**✓**	**✓**	**✓**	**✓**
42	U	Erucic acid (C22:1)	**✓**	**✓**	**✓**	**✓**	**✓**	-
43	S	Behenic acid (C22:0)	**✓**	**✓**	**✓** (a)	**✓**	**✓**	-
44	DH	11,12-dihydroxyeicosanoic acid	**✓**	**✓**	-	**✓**	**✓** (b)	-
45	DH	11,12-dihydroxyeicosanoic acid	**✓**	**✓**	-	**✓**	**✓** (b)	-
46	S	Tricosanoic acid (C23:0)	-	-	-	-	**✓** (b)	**✓**
47	R	15-Hydroxy-7-oxo-dehydroabietic methyl ester	**✓**	**✓**	**✓** (a)	**✓**	**✓**	**✓**
48	R	15-Hydroxy-7-oxo-dehydroabietic acid	**✓**	**✓**	**✓**	**✓**	**✓** (b)	**✓**
49	U	Nervonic acid (C24:1)	**✓**	**✓**	-	-	-	-
50	S	Lignoceric acid (C24:0)	**✓**	**✓**	**✓**	**✓**	**✓**	**✓**
51	DH	13, 14—dihydroxydocosanoic acid	**✓**	**✓**	-	**✓**	**✓** (b)	-
52	DH	13, 14—dihydroxydocosanoic acid	**✓**	**✓**	-	**✓**	**✓** (b)	-
53	S	Cerotic acid (C26:0)	-	-	**✓** (b)	**✓**	**✓** (b)	**✓**
54	St	Cholesterol	**✓**	**✓**	-	-	**✓**	**✓**
55	S	Montanic acid (C28:0)	-	-	**✓** (b)	-	-	-
56	St	β-sitosterol	**✓**	**✓**	-	-	**✓**	-
57	S	Melissic acid (C30:0)	-	-	-	-	-	-

*—labels “a” and “b” denote two different sampling points extracted from the same animal mummy.

**Table 3:** Criteria used to distinguish the presence of ruminant animal fat based on fatty acid ratios of sum peak areas.

**(C15:0 + C17:0)/** **(C12:0 + C14:0 + C16:0 + C18:0)**	**Samples**					
	**90001291**	**90002282**	**90001211**	**90002283**	**90002284**	**90010003**
	0.01	0.01	0.06	0.09	0.01	0.07

**Figure 3:**
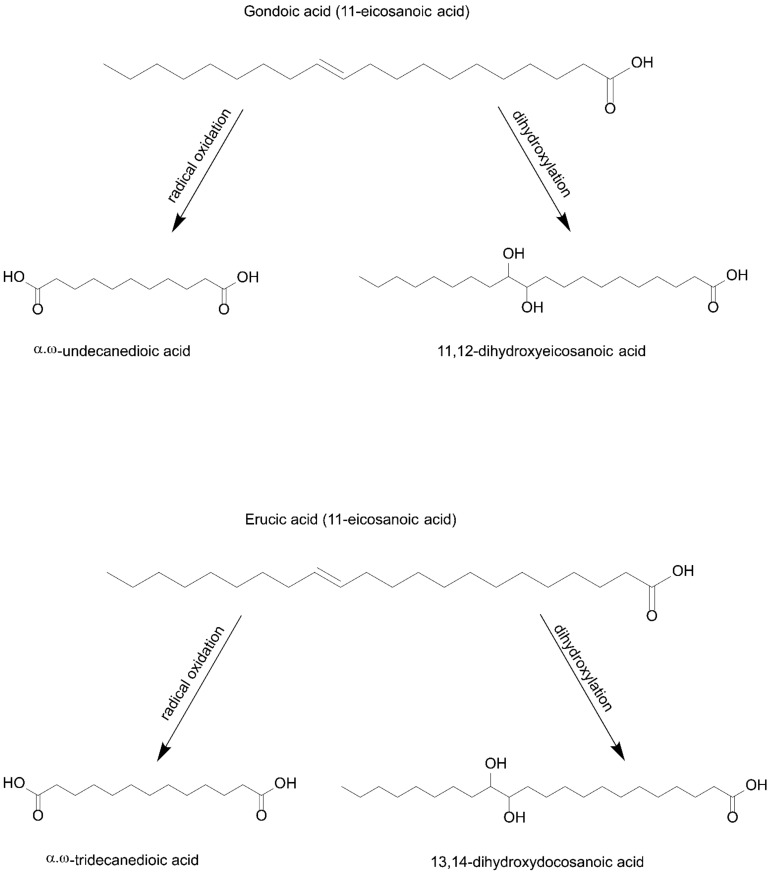
Degradation/alteration pathways of gondoic and erucic acids.

**Figure 4:**
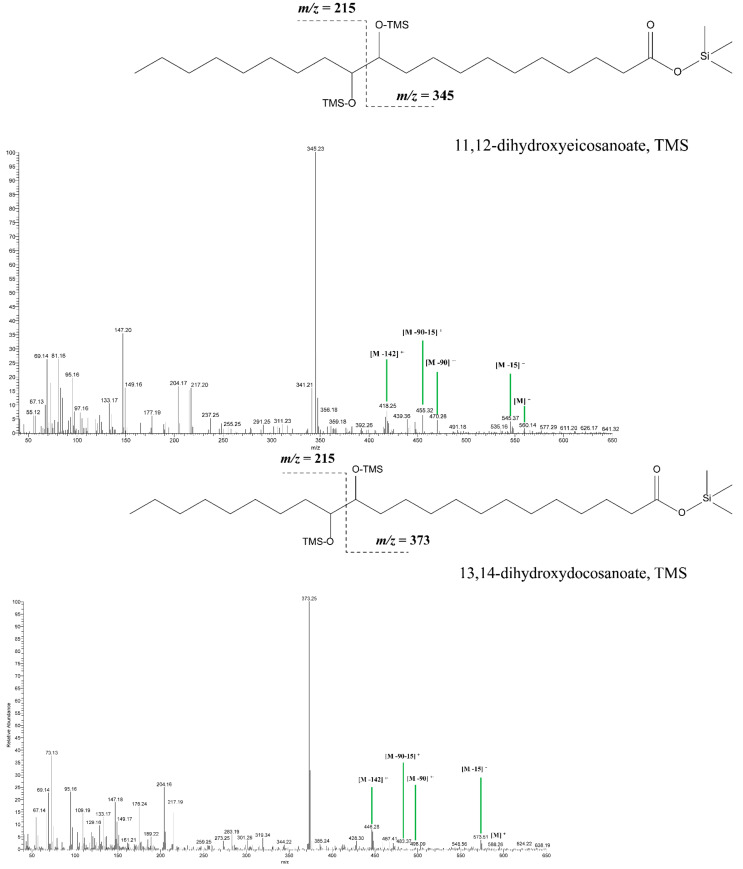
Mass spectra of threo/erythro isomers of 11,12-dihydroxyeicosanoate and 13,14-dihydroxydocosanoic acid.

In this regard, based on the presence of characteristic and diagnostic biomarkers in chromatograms of five gazelle mummies (90001291, 90002282, 90001211, 90002283, and 90002284), the authors of this study present the results of research that, for the first time, inform the scientific community about the use of cruciferous oil derived from the seeds of plants from the Brassicaceae family in the context of animal mummification in Roman Egypt during the 1st to 4th century AD. Indeed, the use of this type of oil obtained from the seeds of Brassicaceae plants has already been documented in several studies conducted by Colombini et al. [3], Copley et al. [4], and Romanus et al. [5] but in the context of fuel necessary for lamp illumination. Furthermore, in ancient historical records, the use of Brassicaceae seed oil as a fuel for ceramic lamps during the Roman period of dominance in ancient Egypt was mentioned by several classical authors (Naturalis Historia by Pliny the Elder, books XV:7 and XIX:26). In their experimental works, it was shown that Brassicaceae oils (i.e., from radish, turnip, mustard, rapeseed) in aged and degraded mixtures were composed of a series of linear monocarboxylic saturated fatty acids (FA), ranging from 6 to 28 carbon atoms with palmitic (C_16:0_) and stearic (C_18:0_) acid as the most prominent, followed by a high abundance of several unsaturated FAs, such as oleic acid (C_18:1∆9_), gondoic acid (C_20:1∆11_) and erucic acid (C_22:1∆13_). The profile was completed by the presence of a series of short-chain *α,ω*-dicarboxylic acids, ranging from 4 up to 12 carbon atoms, with azelaic (nonanedioic acid), sebacic (octanedioic acid), and suberic (decanedioic) acids as the main constituents of this group of molecules, followed by three long-chain dihydroxycarboxylic acids with 18, 20 and 22 carbon atoms, namely, 9,10-dihydroxyoctadecanoic acid, 11,12-dihydroxyeicosanoic acid and 13,14-dihydroxydocosanoic acid as a pair of threo-erythro isomers, hence serving as diagnostic oxidation and dihydroxylation markers for this type of commodity [3,4,5]. That being said, in chromatograms of gazelle mummies 90001291, 90002282, 90001211, 90002283, and 90002284, each of these compounds were found as can be observed in the corresponding figure (Figure 2) and table (Table 2), thus indicating the use of seed oil derived from the Brassicaceae plant family. In all of the samples, a very distinctive fatty acid profile characterized by the presence of extremely high values for oleic, gondoic, erucic and long straight-chain saturated fatty acids such as eicosanoic (C_20:0_) and docosanoic (C_22:0_) acids, was observed. It is important to note that in both fresh and degraded oils derived from the seeds of this plant family, in corresponding chromatograms, values for erucic acid are always significantly higher when compared to gondoic acid, which was the case with the animal mummies that were the focus of this research work. Bearing in mind that gondoic and erucic acids are unsaturated in nature, in burial conditions, they will be susceptible to radical oxidation and dihydroxylation processes, due to the presence of the double bond (Figure 3). Moreover, these unsaturated fatty acids in burial conditions gave rise to the formation of *α,ω*-undecanedioc, and *α,ω*-tridecanedioic acids together with threo and erythro isomers of 11,12-dihydroxyeicosanoate (M°^+^ 560; base peak *m/z* 345) and 13,14-dihydroxydocosanoic acid (M°^+^ 588; base peak *m/z* 373), which are visible as two vicinal peaks in all of the chromatograms related to gazelle mummies containing Brassicaceae seed oils (Figure 4). The presence of these dihydroxycarboxylic acids is consistent with their formation via radical oxidation reactions involving the dihydroxylation of the double bond [3,4,5]. That being said, the mass spectra of these dihydroxycarboxylic acids are characterized by fragment ions arising from the α cleavage of the bond between the two vicinal trimethylsiloxy groups with characteristic abundant fragments at *m*/*z* 215 and *m*/*z* 345 for 11,12-dihydroxyeicosanoic acid, and at *m*/*z* 215 and *m*/*z* 373 for 13,14-dihydroxydocosanoic acid [3,4].

**Figure 5:**
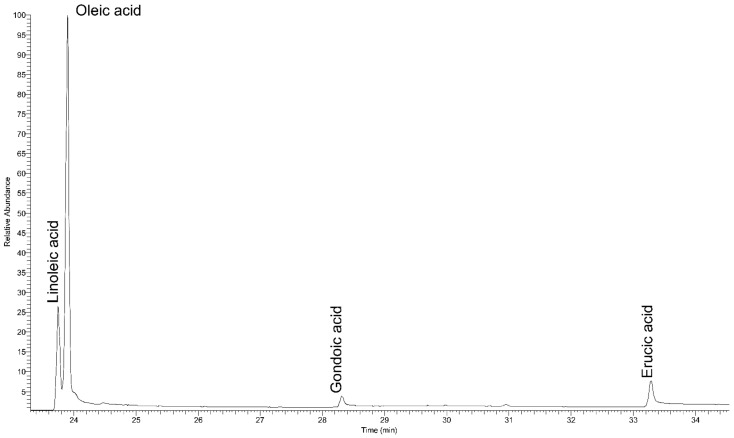
Total ion current chromatogram of the acidic fraction of fresh radish oil.

Therefore, given the fact that these vicinal dihydroxy acids (as threo-erythro isomers) formed through dihydroxylation of the double bond of gondoic and erucic acids in the original balm, as well as the fact that these two unsaturated FAs are present only in cruciferous oils derived from the seeds of certain Brassicaceae plants, these results can be accepted as chemical evidence for the use of this type of natural substance in the context of animal mummification in Roman Egypt, most probably rapeseed or radish oil, which would be in accordance with historical records of that particular time period [39]. Furthermore, the analysis of commercial fresh oil obtained from the seeds of radish plants served as an additional argument in the previously stated claim. As can be seen in Figure 5, in the corresponding TIC chromatogram, the main components of fresh radish oil are linoleic, oleic, gondoic, and erucic acid. Moreover, on the corresponding chromatogram, a feature can be observed that is characteristic only for cruciferous oils obtained from the seeds of plants of the Brassicaceae family, which is that the values of erucic acid are higher compared to those of gondoic acid (also present in aged archaeological samples such as mummification balms). An extensive study of the chemical characterization of fresh oil obtained from the Brassicaceae family of plants, followed by thermo-oxidative treatments (to induce accelerated aging) and the corresponding products that arose, can be found in Colombini et al. [3].

Given the fact that the detection of 9,10-dihydroxyoctadecanoic acid is of a little diagnostic value except as evidence for the presence of oleic acid (C_18:1∆9_) in the original balm, it is not feasible to identify the botanical origin of this molecule solely and its directly related compounds, considering that oleic acid is a universal constituent of both plant oils and animal fats. On the other hand, extremely high values of C9 (azelaic) diacid and oleic acid, together with the aforementioned dihydroxy acid, in samples 90001291 and 90002282, it is possible to argue about the possibility of the presence of plant oil (olive, linseed oil, etc.) rich in oleic acid used for mummification of these two specific gazelle mummies.

Dicarboxylic acids are not naturally present in plant oils, resins, waxes, and animal fats; they are formed as a result of several different oxidative degradation mechanisms of the double bond(s) in mono-, or poly-unsaturated fatty acids. Thus, the position of the double bond is directly related to the carbon chain length of the diacids formed. Therefore, the presence and the amount of diC9 (azelaic acid, originating from C18 fatty acid with a double bond between carbons 9 and 10 like linoleic and linolenic acids) combined with the presence of diC8 (suberic acid) and diC7 (pimelic acid) acids are indicators of degraded vegetable oils, thus suggesting that a siccative or semi-siccative plant oil high in oleic acid was used during mummification.

#### 2.2.2. Presence of Beeswax

Due to its complex chemical composition (hydrocarbons, mono/di/triesters, hydroxypolyesters, free acids, and free alcohols), archaeological beeswax requires a prior saponification process in order to be able to analyze it with GC-MS apparatus in the first place. The most common approach is alkaline saponification in hydroalcoholic potassium hydroxide, which allows us to break the ester bond and, moreover, to separate the saponifiable fraction (acids) and an unsaponifiable fraction (alcohols, sterols, and hydrocarbons). The use of beeswax as an embalming agent has been already well documented in numerous archaeometric studies concerning ancient Egyptian mummification of both humans and animals [6,7,9,14,15,16,40]. That being said, in all the gazelle mummies, except for sample 90002284, the presence of long carbon even-chained saturated monocarboxylic fatty acids ranging from C_22:0_ to C_30:0_ with a maximum at C_24:0_, together with the occurrence, in saponified extracts, of long carbon even-chain alcohols consisting of 24 to 32 carbon atoms, confirmed the presence of beeswax as another essential ingredient used as mummification substance (Table 2 and Table 4).

**Table 4:** List of identified fatty alcohols obtained from saponification of embalming substances from gazelle mummies.

Identified Compounds	Samples					
	90001291	90002282	90001211 *	90002283	90002284	90010003
Tetracosanol (C24)	**✓**	**✓**	**✓** (a)	**✓**	-	**✓**
Hexacosanol (C26)	**✓**	**✓**	**✓**	**✓**	-	**✓**
Octacosanol (C28)	**✓**	**✓**	**✓**	**✓**	-	**✓**
Triacontanol (C30)	**✓**	**✓**	**✓**	**✓**	-	**✓**
Dotriacontanol (C32)	**✓**	**✓**	**✓** (a)	**✓**	-	-

*—labels “a” denote a different sampling point extracted from the same animal mummy.

The identified alcohols in beeswax from an archaeological context are normally an indicator of degradation. Although the presence of odd chain n-alkanes was not observed in corresponding chromatograms, the most probable cause is that the loss of alkanes was due to the pre-heating of the original beeswax during use in order to be able to mix it with other ingredients of the original embalming substance.

#### 2.2.3. Presence of Conifer Resin

Results of the analysis by GC-MS revealed that one of the ingredients used for embalming was from natural resins extracted from the Pinaceae family of trees. Further data assessment confirmed the presence of resins in samples through the identification of dehydroabietic acid (M°^+^ 372; base peak *m/z* 239) as its most dominant diterpenoid constituent together with its oxidation products: 3-hydroxydehydroabietic acid (M°^+^ 460; base peak *m/z* 191), 7-hydroxydehydroabietic acid (M°^+^ 460; base peak *m/z* 237), 15-hydroxydehydroabietic acid (M°^+^ 460; base peak *m/z* 327), 7-oxo-dehydroabietic acid (M°^+^ 386; base peak *m/z* 253) and 15-hydroxy-7-oxo-dehydroabietic acid (M°^+^ 474 base peak *m/z* 459), which are formed from the degradation of abietadienic acids, present in fresh diterpenic resin of conifer trees, due to aging (markers of natural degradation over time) [2,7,26,38,41,42]. Moreover, in the studied samples, and directly related to the presence and preparation methods of resins as one of the ingredients for embalming, the presence of retene (final stable end product of thermal degradation of diterpenoids from Pinaceae resin), and methyl esters of dehydroabietic acid (M°^+^ 314; base peak *m/z* 239), 7-oxo-dehydroabietic acid (M°^+^ 328; base peak *m/z* 253) and 15-hydroxy-7-oxo-dehydroabietic acid (M°^+^ 416; base peak *m/z* 401) was observed. Therefore, the occurrence of this aromatized molecule (retene) together with the presence of the aforementioned methyl esters (these derivatives are otherwise absent when the resin is heated alone) in studied samples indicates that the resin was exposed to an anthropogenic thermal treatment involving strong and destructive distillation of resinous Pinaceae wood in order to produce the protective substance named wood tar. In addition to diterpenoids with abietane skeletons, compounds with pimarane carbon structures were also detected in several gazelle mummies: pimaric (M°^+^ 374; base peak *m/z* 121), sandaracopimaric (M°^+^ 374; base peak *m/z* 121) and isopimaric acid (M°^+^ 374; base peak *m/z* 241). Indeed, these molecules could be indicative of the presence of sandarac resin extracted from the Cupressaceae family of trees, however, with no traces of diagnostic and characteristic compounds, such as agathic, trans-communic, and hydroxysandaracopimaric acids, that hypothesis was not considered.

To summarize, all sample extracts from six gazelle mummies showed insignificant to no difference at all regarding the presence of biomarkers characteristic for diterpenic compounds. As already described and can be seen in Table 2, all samples showed the presence of markers of natural degradation (dehydroabietic acid, 3-hydroxy-dehydroabietic acid, 7-hydroxy-dehydroabietic acid, 7-oxo-dehydroabietic and 15-hydroxy-7-oxo-dehydroabietic acids). Markers of anthropogenic modification were also present. Retene suggested intense heating of resin from the Pinaceae family. In the end, methylated diterpenic acids such as 7-oxo-dehydroabietic methyl ester and 15-hydroxy-7-oxo-dehydroabietic acid methyl ester indicated that this ingredient was obtained by pyrolytic treatment of resinous pine wood. Considering that resins and resinous wood are hard and solid materials in their natural form, in order to use their antibacterial and waterproofing properties for mummification practices, they had to be exposed to strong and long heating treatments as well as to processes involving distillation, thus altering the chemical composition of the original raw material, thus making the medium more flexible and easily applicable, especially when combined with plant oils.

## 3. Materials and Methods

All nine samples were extracted from six gazelle mummies using a clean scalpel that was decontaminated before and after each sampling procedure. Afterwards, the extracts were deposited and stored in glass vials.

All solvents and reagents were of analytical grade. Tetrahydrofuran, hexane, and N,*O*-Bis(trimethylsilyl)trifluoroacetamide/Trimethylchlorosilane (BSTFA/TMCS) were supplied by Sigma-Aldrich. Ethanol, dichloromethane, and diethyl ether were supplied by Merck. Given the preservation state of the archaeological objects in focus, it was possible to obtain samples from several different points within individual gazelle mummies, which further allowed additional data assessment related to processing possible variabilities in the composition of the embalming substance within a single gazelle, and, of course, to address the question of mummification of these animals within the whole context. The general sampling strategy was realized in accord with the European Standard EN 16085 resolution. Owing to the heterogeneous composition of balms constituting several natural substances in a different stage of alteration/degradation, averaged samples were analyzed. The total amount of each sample was crushed into fine powder to obtain these.

### 3.1. Description of Archaeological Samples

**Figure 6:**
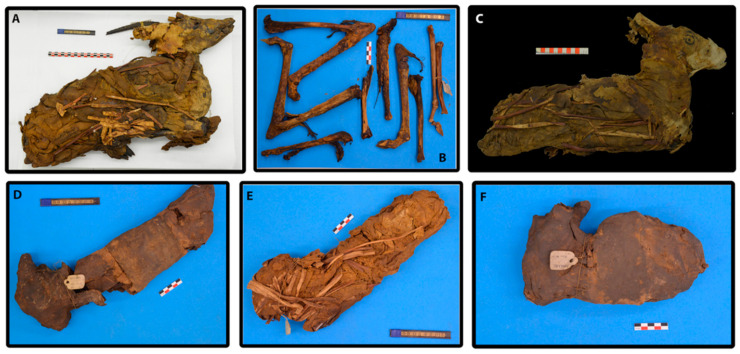
The set of six animal mummies from the Confluence Museum: (**A**) gazelle mummy, Inv 90001211; (**B**) gazelle mummy, Inv 90010003; (**C**) gazelle mummy, Inv 90001291; (**D**) gazelle mummy, Inv 90002284; (**E**) gazelle mummy, Inv 90002283; (**F**) gazelle mummy, Inv 90002282 (© Projet MAHES).

The mummies analyzed in this study came from nineteenth century excavations that were carried out by Gaston Maspero in 1882 in Kom-Mereh near Esna. Collected animal mummies have been transported to France and kept at Confluence Museum in Lyon since 1903. In this regard, given the immeasurable value of the cultural heritage that these archaeological relics represent, the six animal mummies that are the subject of this study, as well as their respective wrappings, were sampled whilst taking care not to compromise the integrity of these patrimonial artefacts (Figure 6). The set consisted of 5 relatively well-preserved mummies that were still wrapped in their original textile (90001211; 90001291; 90002282; 90002283, 90002284), and leg fragments of a destroyed mummy whose textiles had been previously removed (90010003). From an archaeozoological analysis, we identified an adult (90010003), a young adult female (90001211), and four juveniles whose sexual gender could not be specified (Porcier & Chaix 2022). Nine samples were collected from the six mummies. Two samples from different locations of the respective gazelle mummies were collected for samples referred to 90001211, 90002282, and 90002284.

For the mummies:

90001211: Two samples (textile) respectively extracted from the exterior part of the right shoulder (a) and from the exterior part of the right side of the chest (b)

90002282: Two textile samples recovered from the bottom middle part of the mummy, light colored sample (a) and dark colored sample (b)

90002284: Two samples respectively extracted from the interior part of the chest area (a) and textile recovered from the head of the mummy (b).

### 3.2. FT-IR

A preliminary assessment was initiated using micro-destructive Fourier transform infrared spectroscopy, where samples (<1 mg) were mixed and homogenized with 150 mg of KBr (VWR International, USA) and then pressed under 10 T/cm^−2^ in order to obtain a KBr pellet. Measurements were performed using a Thermo-Nicolet iZ10 FT-IR spectrometer (Illkirch-Graffenstaden, France) in transmission mode with a deuterated triglycine sulfate (DTGS) detector with OMNIC software. Three FT-IR spectra were performed per sample, and due to the repeatability/reproducibility of the obtained results, a representative spectrum per sample was presented in this study. All FT-IR spectra were collected in the middle infrared region (400 to 4000 cm^−1^) recording 32 scans at 4 cm^−1^ spectral resolution. No spectral pre-processing was performed on the spectra. The presence of both inorganic and organic matter was confirmed through observation of diagnostic vibrational group frequencies and comparison with reference data (Table 5) [14,15,17,24,26,43].

### 3.3. GC-MS

All samples were analyzed by gas chromatography mass spectrometry. Samples (10 mg of organic matter in powdered form) were solvent-extracted using 1 mL of dichloromethane three times followed by ultrasound (10 min) and centrifugation (6000 rpm for 10 min), after which the supernatants were combined and dried under a gentle stream of nitrogen. Dried extracts were derivatized with 200 μL of BSTFA/TMCS (99/1 *v/v*) then heated at 70 °C for 30 min, followed by second evaporation, redissolved in 1 mL of hexane/dichloromethane mixture (1/1, *v/v*), then filtered on a polytetrafluoroethylene cartridge (PTFE, 0.45 μm, VWR). One microliter of the solution was injected into the GC-MS apparatus. GC-MS was performed with a Thermo Scientific Focus gas chromatographic system composed of a Thermo Scientific Al 3000 auto-sampler coupled with an ITQ 700 ion trap mass spectrometer (Thermo Fisher Scientific, Illkirch-Graffenstaden, France). A GC fused silica capillary column Thermo trace GOLD TG-5MS (5% diphenyl/95% dimethylpolysiloxane, 30 m length × 0.25 mm i.d. × 0.25 μm film thickness) was used. The carrier gas was helium with a constant flow of 1 mL.min-1. One microliter of each sample was injected in splitless mode. Mass spectra were recorded in electron impact mode with an electron ionization voltage of 70 eV, an ionization time of 25,000 μs, and a mass range of 40–650 *m/z*. The injector temperature was set at 250 °C. Transfer line, ion trap and manifold temperatures were respectively set at 300 °C, 200 °C and 50 °C. The separation was achieved with the following temperature program: 50 °C with a 2 min hold, increased by 10°C min^−1^ to 200 °C, then increased by 2.5 °C min^−1^ to 310 °C, then increased by 8°C min^−1^ to 330 °C and held isothermally for 3 min. All of the injections were realized in triplicate [44]. The peak assignments were realized based on comparison with retention times and mass spectra of pure standard compounds and by using the NIST database (NIST MS Search 2.0). Samples were additionally analyzed through saponification and solid phase extraction in order to observe the possible presence of characteristic biomarkers of beeswax (saponification: long-chain fatty acids, fatty alcohols, palmitic wax esters, etc.) and diagnostic saturated hydrocarbons (solid-phase extraction: steranes and hopanes) unique for bitumen.

#### 3.3.1. Saponification

Samples (10 mg in powdered form) were extracted with 3 × 1 mL of THF followed by an ultrasound bath (5 min) and centrifugation at 6000 rpm (5 min). The solvent extracts were combined, and then 2 mL of a solution of potassium hydroxide KOH 10% in MeOH/H_2_O (9/1, *v/v*) was added. The mixture was magnetically stirred and heated at 65 °C for 1 h. After evaporation, 3 mL of pure water were added with 1 mL of HCl 5 M. The aqueous phase was washed with 3 × 5 mL of diethyl ether. The organic phases were combined and dried with anhydrous sodium sulphate, then filtered on filter paper. Excess reagent was evaporated to dryness under a stream of nitrogen. Trimethylsilylation was applied. After evaporation, the derivatized sample was solubilized in hexane/DCM mixture (2/1, *v/v*) and filtered on a PTFE cartridge before injection in GC–MS [45].

#### 3.3.2. Solid Phase Extraction

Samples (10 mg in powdered form) were extracted with 1 mL of hexane/THF (1/1; *v/v*) aided by ultrasound for 5 min and then centrifuged at 6000 rpm for 5 min. The supernatant was set aside in order to extract the solid pellet again, and these steps were repeated 3 times. The three fractions were combined and evaporated to dryness under a stream of nitrogen, then redissolved with 500 μL of hexane/THF (1/1; *v/v*). This solution is called charging. In parallel, a Strata^®^ 200 mg/3 mL SPE cartridge (Phenomenex, Torrance, CA, USA) was calibrated with 4 mL of hexane. The initial 500 μL charge was deposited on the cartridge, then a first elution was carried out with 4 mL of hexane and collected in a hemolysis tube (fraction 1 for presence or absence of markers for bitumen, such as hopanes and steranes). A second elution was carried out with 4 mL of EtOH and then collected in another tube (fraction 2 for the presence of resins). A final elution was carried out with 4 mL of DEE and 2% AcOH (fraction 3 for the presence of fatty acids). Each fraction was evaporated to dryness under a stream of nitrogen. After trimethylsilylation, fractions 1 and 2 were solubilized with 60 μL of a hexane/DCM mixture (2/1; *v/v*), while fraction 3 was solubilized with 1.5 mL of hexane/DCM (2/1; *v/v*). The three fractions were then filtered on a PTFE cartridge (0.45 μm) before respective injections in GC–MS apparatus [46].

## 4. Concluding Remarks

Molecular characterization of embalming materials used in the mummification of six gazelle mummies found at the Roman archaeological site of Kom Mereh in today’s modern Egypt was performed. Analysis revealed the use of four distinct families of material as essential ingredients in the formulation of embalming substances (Table 6).

Plant oils and animal fats were identified in all nine sample extracts recovered from six gazelle mummies, and data, both infrared and chromatographic, suggested that no major or significant discrepancies between different sampling points within individual gazelles were present, thus implying that identical, or extremely similar, plant oil and/or animal fat was used as one of the ingredients for the mummification of the whole body of the animal. This result itself is noteworthy because when compared to human mummification practices, it is known from written classical records and archaeometric studies that in human mummies, one can recognize different balms used to mummify the head of the deceased, while the body of the same individual would be coated with substantially different embalming substances. Furthermore, all nine sample extracts among six gazelle mummies showed considerably similar fatty acid profiles (presence of fatty acids ranging from C_6:0_ to C_18:0_), thus more specific determination of the origin of plant oil and/or animal fat was not possible at that time, meaning future studies will have to shed light on this question (i.e., the use of isotope ratio combustion mass spectrometry for identifying the origin of animal fat). In the case of animal mummies from Kom Mereh, the only exception to this rule would be the presence of cruciferous oils derived from the seeds of the Brassicaceae botanical family (in this case, most probably oil from radish seeds) detected in a total of five gazelle mummies (90001291, 90002282, 90001211, 90002283 and 90002284) according to the presence of characteristic and diagnostic biomarkers (gondoic and erucic acids and their respective oxidative and dehydroxylated compounds). Given the case that in the scientific literature, this is the first acknowledgment of the use of this type of commodity in animal mummification, the question that arises is in what capacity was this oil used as one of the ingredients in the formulation of the embalming material? The answer lies in the fact that in Roman Egypt, Greeks and Romans intentionally mixed Brassicaceae oils with other types of materials (such as beeswax and/or resins, etc.) when they produced famous funerary mummy portraits (portraits of individuals painted on the coffins in which they were buried) [47]. As they were already familiar with the results of mixing different oils with beeswax, hence allowing the newly formed product to be more workable and adaptable, we can assume that in the case of gazelle mummies from Kom Mereh, workers applied this knowledge when they were preparing the embalming substance for later mummification.

In this regard, a total of five animal mummies contained beeswax most likely to originate from *Apis melifera*, the most common North African honeybee that was harvested for a long period of time in ancient Egypt for its products [47]. What is possible to realize based on data published by several authors and the results obtained in this study is that beeswax in the Roman period of rule in ancient Egypt was imposed as a kind of necessity in mummifying different types of animal mummies (votive, sacred, visceral, pets, etc.) primarily because of its preferable properties against body degradation and positive reaction when mixed with heated resins and plant oils/animal fats.

Regarding the presence of diterpenoid markers, both FT-IR and GC-MS undoubtedly reported the presence of diterpenic resins extracted from the Pinaceae family, most probably from pine trees. Coniferous trees were not locally grown in ancient Egypt; thus, to be able to use resins from this family of plants in producing mummification balms, this material would have to be imported from Mediterranean lands, especially during Roman times when this practice was intensified. Furthermore, as in the previous case with plant oils/animal fats, no significant differences in the presence and concentration of identified compounds were detected between different sampling points within individual gazelles, thus implying that the same type of diterpenic resin was used in mummifying the whole body of the animal.

Regarding the presence of bitumen in analyzed samples extracted from all six gazelle mummies, the main diagnostic chemical families (saturated hydrocarbons such as n-alkanes, linear and/or branched; polyaromatic hydrocarbons; tetracyclic saturated hydrocarbons, steranes; pentacyclic terpenoids, hopanes) in this substance were not detected [8,48,49].

The presence of natron or lime was observed through FT-IR data analysis of samples from two gazelle mummies, namely 90002282 and 90002283. That being said, we can acknowledge that the two aforementioned gazelle mummies differed from the others in terms of the use of inorganic agents in addition to existing organic ones.

In addition to this, we also noticed that mummy number 90010003 was completely different from the others in terms of the absence of markers characteristic of oils derived from the cruciferous seeds of Brassicaceae plants. This information is not surprising given the state of preservation of said mummy, where only naked bones remained preserved (Figure 6). Despite these few obvious differences, we can assume that all six gazelle mummies from the Roman period Kom Mereh were mummified in a similar manner with the same choice of embalming agents.

To conclude, the results of this study showed that the applied embalming substances were complex mixtures consisting of plant oils and/or animal fats, resins, and beeswax (both local and imported) during the Roman period of dominance in ancient Egypt, and when compared to results from other archaeometric studies dealing with the study of animal mummification [6,7,8,40,50], it can be deduced that similar choices of available raw materials were used on other types of animals, such as the case with ibis mummies from the works of Buckley et al. (2004), Brettell et al. (2017) and Markovic et al. (2020).

## Figures and Tables

**Table 1:** FT-IR interpretation and initial data assessment (ν—stretching vibration; δ—bending vibration; ρ—rocking mode).

90001291	90002282	90001211	90002283	90002284 *	90010003	Assignment Bond	Source
3393	3416	3412	3413	3414	3412	ν OH	Oils, resins
3283				3289 (a)		ν OH	Oils, resins
3070				3070 (a)		ν (C=C)	Diterpenoids
2964	2959			2954 (a)		ν CH_3_	Resins
2926	2920	2928	2920	2927	2926	ν CH_2_	Oils/fats, resins, beeswax, bitumen
2872	2871			2871 (a)		ν CH_3_	Resins
2854	2851	2856	2851	2854	2852	ν CH_2_	Oils/fats, resins, beeswax, bitumen
				2636 (a)	2644	ν OH	Diterpenoids
	2519		2515			νOH	CaCO_3_
1715	1710	1712	1710	1709	1712	ν (C=O)	Resins
1653	1652			1655 (a)		ν (C=O)	Resins
		1609	1624	1637 (b)	1637	ν (C=C)	Polysaccharides
1541	1552		1546	1546 (a)		δ (CH_3_) Amide II	Organic material, Proteins
1454	1460	1460	1463	1453	1462	δ (CH_2_)	Oils/fats, resins, beeswax, bitumen
1413	1427		1413		1411	ν CO_3_	Organic material, CaCO_3_
1384	1383	1383	1383	1384	1384	δ (CH_3_)	Oils/fats, resins, beeswax, bitumen
1318	1319	1315	1318	1314	1316		Calcium oxalates
		1283	1281			ν (C-O)	Resins
1243	1248	1237	1236	1240	1240	ν (C-O)	Diterpenoids
1175	1163	1169	1161	1171	1181	ν (C-O)	Oils/fats, resins, beeswax
				1127 (a)		ν (C-O)	Organic material
1107	1112	1112	1111	1111 (b)	1109	ν (C-O)	Organic material
	1055	1057	1057	1058 (b)	1048	ν (C-O)	Organic material
1041	1032	1032	1031	1040		ν (C-O)	Organic material
	874		872				CaCO_3_
		839		823 (a)		ρ (C-H)	
720				722 (a)	720	ρ (C-H)	Oils/fats, beeswax
	712		711				CaCO_3_

*—labels “a” and “b” denote two different sampling points extracted from the same animal mummy.

**Table 5:** Reference bibliography used for comparison.

Nature of Samples	Reference Bibliography
Plant oil	[43]
Animal fat	[43]
Pinaceae resins	[24,26,43]
Beeswax	[43]
Natron	[14,15,17]

**Table 6:** Chemical composition of the studied balms.

No. Samples	Findings
90001291	Plant oils (Brassicaceae seed oil), animal fat, Pinaceae resin, wood tar, beeswax
90002282a	Plant oils (Brassicaceae seed oil), animal fat, Pinaceae resin, wood tar, beeswax
90002282b	Plant oils (Brassicaceae seed oil), animal fat, Pinaceae resin, wood tar, beeswax
90001211a	Plant oils (Brassicaceae seed oil), animal fat, Pinaceae resin, wood tar, beeswax
90001211b	Plant oils (Brassicaceae seed oil), animal fat, wood tar, beeswax
90002283	Plant oils (Brassicaceae seed oil), animal fat, Pinaceae resin, wood tar, beeswax
90002284a	Plant oils (Brassicaceae seed oil), animal fat, Pinaceae resin, wood tar
90002284b	Plant oils (Brassicaceae seed oil), animal fat, Pinaceae resin, wood tar
90010003	Plant oils, animal fat, Pinaceae resin, wood tar, beeswax

## Data Availability

Data presented in this study are available on request from the corresponding author. Data are not publicly available due to a privacy policy of the authors’ institution.

## Sample Availability

Samples are not available.
